# Synthesis and Adsorption Properties of 4-Vinylpyridine and Styrene Copolymer In Situ Immobilized on Silica Surface

**DOI:** 10.1186/s11671-017-1991-2

**Published:** 2017-03-23

**Authors:** E. S. Yanovska, L. O. Vretik, O. A. Nikolaeva, Y. Polonska, D. Sternik, O. Yu. Kichkiruk

**Affiliations:** 1 Taras Shevchenko Kyiv National University, 12 L. Tolstogo Str., 01033 Kyiv, Ukraine; 2 Maria Curie-Skłodowska University, Maria Curie-Sklodowska Sq., 20-031 Lublin, Poland; 3 Ivan Franko Zhytomyr State University, 42 Pushkina Str., 10008 Zhytomyr, Ukraine

**Keywords:** Adsorption, 4-Vinylpyridine-styrene copolymer, Composite, In situ immobilization, Heavy metals, Silica gel

## Abstract

Copolymer of 4-vinylpyridine with styrene was in situ immobilized on silica gel surface via the heterogeneous radical polymerization. Anchorage of the copolymer on the surface layer was confirmed by IR spectroscopy. The quantity of copolymer on the silica gel surface was evaluated as 25.73 wt.% by TG and DSC-MS analysis. “Islet” location of polymer layer on the silica surface was confirmed by the scanning electron microscopy. A high adsorption activity of silica gel with immobilized copolymer towards microquantitatives of Cu(II), Cd(II), Pb(II), Fe(III), and Ni(II) ions in steady state conditions as well as of Ni(II) ions in dynamic regime was found.

## Background

Huge amount of waste water produced annually in the world, makes a problem of efficient methods of water cleaning very important. The adsorption is one of the modern methodology of waste water treatment. The major requirements for the optimal adsorbent are a combination of environmental friendliness, low cost, and efficiency. From this point of view, organic composite materials consisting of natural inorganic carrier (e.g., porous alumosilicate and organic modifier immobilized on its surface) are promising adsorbents for the removal of toxic compounds from the water. Polymer materials with ion-exchange and complexing properties have been tried recently [1–4] as inorganic carrier modifiers. These polymeric modifiers have higher adsorption capacity towards ions of toxic metals than the corresponding monomers [5, 6].

Taking into account, that the good adsorption properties exhibited composites based on nitrogen-containing polymers, e.g., polyhexamethylene guanidine and their derivatives [1, 5, 6], polyaniline [7], we have considered to hold poly-4-vinilpyridine immobilization. Homopolymer of 4-vinylpyridine is water-soluble. That is why we decided to synthesize its copolymer with such a hydrophobic and cheap monomer as styrene.

Before immobilization of a new copolymer on the surface of natural carrier it would be useful to investigate its adsorption properties after immobilization on the surface of synthetic porous materials with well-defined properties. In the presented study, silica gel was selected as such model porous carrier [8, 9].

The presented paper deals with syntheses of in situ immobilization on silica gel surface copolymer of 4-vinylpyridine with styrene as well as its adsorption characteristics with respect to Cu(II), Cd(II), Pb(II), Mn(II), Fe(III), and Ni(II) ions.

## Methods

### Synthetic procedure and characterizations

Styrene (Merck) and 4-vinylpyridine (reagent grade, Ukraine) were distilled under vacuum and stored under argon at 3–5 °C. The reagent grade 2,2′-azobis(2-methylpropionitrile) (AIBN, Ukraine) served as an initiator of their radical polymerization. Silica gel (fraction of 0.1–0.2-mm diameter particles, specific surface 428.61 m^2^/g, Merck) was used as inorganic carrier. Other chemicals of p.a. quality (Sigma-Aldrich Inc.) were used as received.

*Composite* was obtained by radical copolymerization of 4-vinylpyridine with styrene in the presence of silica gel. Namely, synthesis of composite was performed as follows: 1.66 g (0.016 mol) of styrene and 2.34 g (0.064 mol) of 4-vinylpyridine with 0.04 g of 2,2′-azobis(2-methylpropionitrile) (AIBN) were dissolved in 16 mL of CCl_4_ and added into the reactor with 20 g of silica gel while stirring. An inert atmosphere was created by argon blowing during 30 min. After the interruption of argon blowing, the reaction mixture was heated to 76 ^o^C. This temperature was maintained during 5 h. Then the reaction mixture was cooled, and the obtained suspension was filtered off. After filtration the suspension was washed with propanol-2 thrice and dried at room temperature during 24 h.

*Solutions with different pH values*. Solution with pH 1.0 was prepared from the standard titrimetric substance of HCl acid. Solutions with pH 1.7 and 4 were prepared from the standard buffers (DSTU 8.135:2009, «RIAP» Ukraine). Alkaline solution with pH 8.4 was prepared by mixing of 17 mL of 1 M acetic acid and 5 mL of 25% ammonia solution and adding distilled water up to 1 l. The pH values of all solutions were controlled by pH-meter.

*FT-IR spectra* of the samples of composite and the silica gel were recorded using an IR spectrometer with Fourier transformation (Thermo Nicolet Nexus FT-IR, USA). The samples were pressed with KBr. The FT-IR spectra were recorded in the spectral range of 600–4000 cm^-1^ with 16 scans per spectrum at a resolution of 4 cm^-1^.

*Thermal analysis.* Thermal analysis was carried out on STA 449 Jupiter F1, Netzsch (Germany) under the following operational conditions: heating rate 10 °C min^-1^, dynamic atmosphere of synthetic air (50 mL min^-1^), temperature range 30–950 °C, sample mass ~18 mg, sensor thermocouple type S TG-DSC. As a reference, empty Al_2_O_3_ crucible was used. The gaseous products emitted during decomposition of material were analyzed by QMS 403C Aeölos (Germany) coupling on-line to STA instrument. The QMS data were gathered in the range from 10 to 160 amu.

*Surface morphology analysis.* The surface morphology of composite was observed by scanning electron microscope (SEM, LEO 1430VP, Carl Zeiss, Germany).

*The investigations of adsorption properties.* The investigations of adsorption properties of the obtained composite with respect to Cu(II), Cd(II), Pb(II), Mn(II), Fe(III), and Ni(II) ions were carried out in the static mode with periodic hand-stirring. For that the sample of 0.1 g of synthesized adsorbent was contacted with 25 mL of nitrate solutions of the abovementioned metals at different concentrations. Nitrate solutions of Cu(II), Pb(II), Mn(II), Fe(III), Ni(II), and Co(II) were prepared with the sets of “standard sample solutions” of these salts on 1 M HNO_3_ background (produced by A.V. Bogatsky FHI, Odesa) with concentrations of 1 and 10 mg/mL.

The adsorption properties of the synthesized composite with respect to Ni(II) ions were investigated in the dynamic mode. 50 mL of solutions of Ni(II) nitrates with 100 μg–1 mg of Ni(II) ions passed through glass columns filled with 0.1 g of adsorbent at the rate 1 mL/min.

Determination of the equilibrium concentration of the metals was carried out by atomic absorption using a flaming atomic absorption spectrophotometer “Saturn” (Ukraine) in “air-propane-butane” flame mixture.

### Calculations

The adsorption capacity (*A*) was calculated using the formula:

$$ A=\left({c}_o-{c}_e\right)\; V/ m; $$

the degree of adsorption (*R*) was calculated using the formula:

$$ R=\left({c}_{ads}/{c}_o\right)\cdotp 100\%=\left({c}_o-{c}_e\right)/{c}_o\cdotp 100\%, $$

where *c*_*o*_ is the concentration of initial solution, *c*_*e*_ is the equilibrium concentration of metal, *V* is the volume of equilibrium solution, and *m* is the mass of adsorbent.

## Results and Discussion

### Physicochemical Characteristics of the Composite

**Fig. 1:**
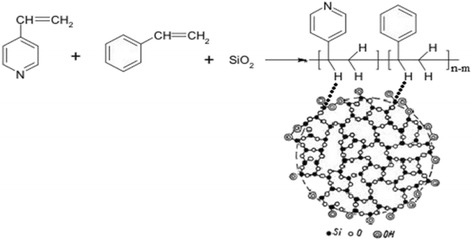
The scheme of in situ immobilization of 4-vinylpyridine and styrene copolymer on silica gel surface

The scheme of in situ immobilization of 4-vinylpyridine and styrene copolymer on silica gel surface is presented in Fig. 1.

**Fig. 2:**
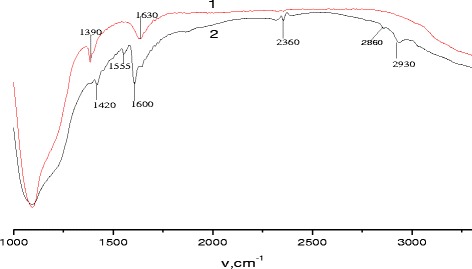
FT-IR spectra of silica gel (*1*) and the synthesized composite (*2*)

Anchorage of the copolymer on the silica gel surface was confirmed by FT-IR spectroscopy. The corresponding spectra of parent silica gel and the synthesized composite are presented in Fig. 2.

Comparative analysis of IR spectra of composite and parent silica gel showed (Fig. 2) that in the spectrum of composite some absorption bands associated with the immobilized copolymer are presented. Absorption bands at 1555 and 1600 cm^-1^ in the spectrum of the composite correspond to the vibrations of double C=C bonds in aromatic rings of 4-vinylpyridine and styrene.

Absorption bands at 2860 and 2930 cm^-1^ could be attributed to the valence vibrations v(CH) in CH_2_ and CH groups of the main polymer chain. Thus, FT-IR spectra gave confirmation of copolymer immobilization on the silica gel surface.

The influence of polymeric coating on thermal properties of silica was studied by TG and DSC-MS analysis. Such methods also were employed in order to determine the mass ratio of copolymer coating on the silica surface.

**Fig. 3:**
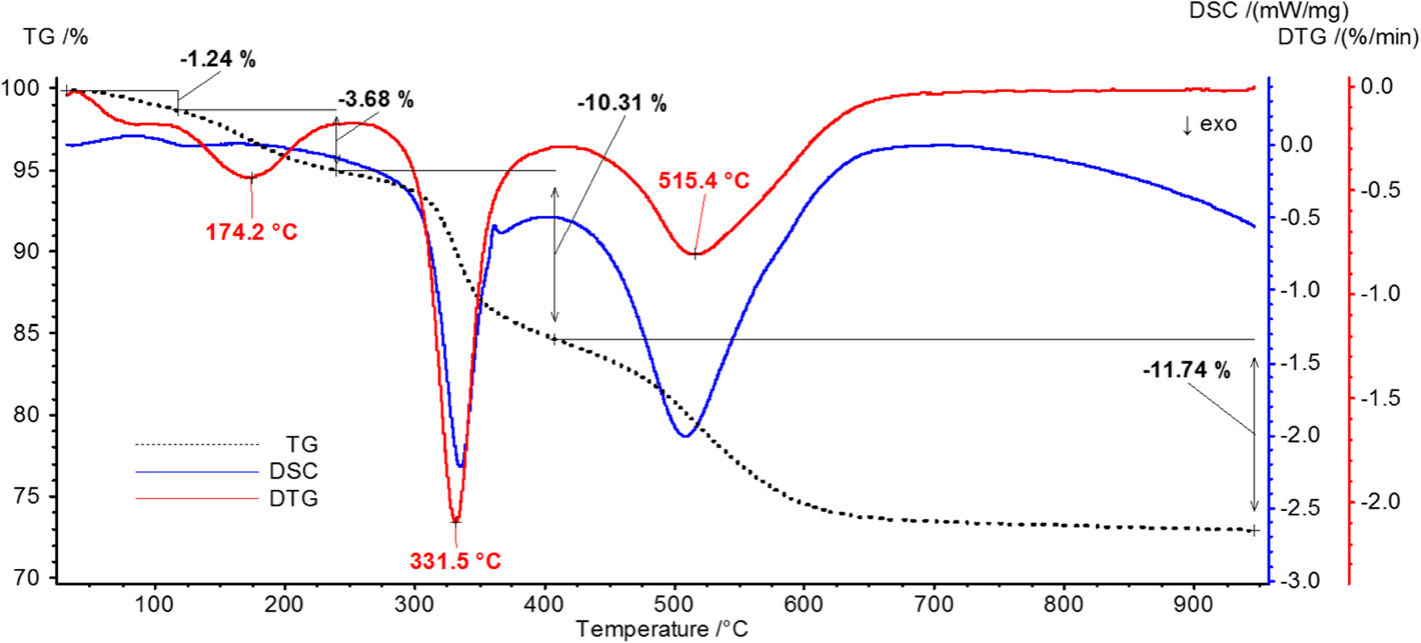
TG, DTG, DSC curves of the synthesized composite

**Fig. 4:**
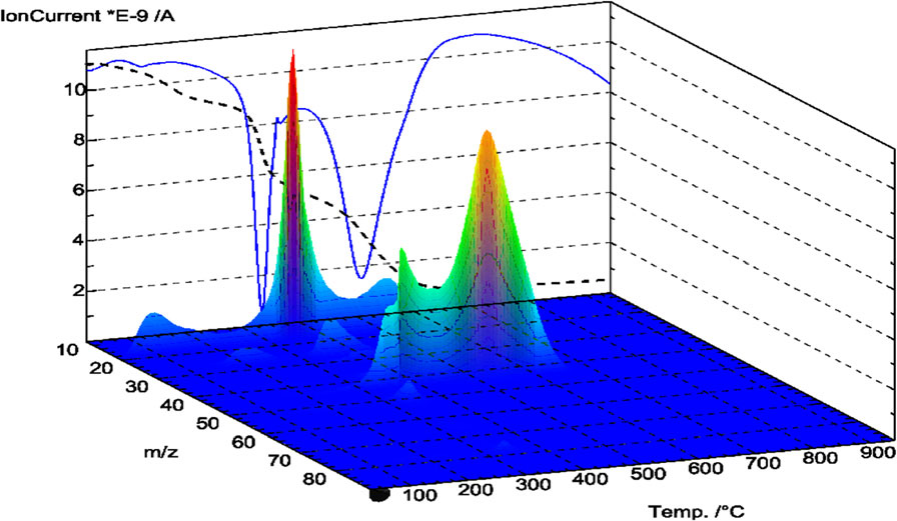
DSC-QMS-3D of the synthesized composite

**Fig. 5:**
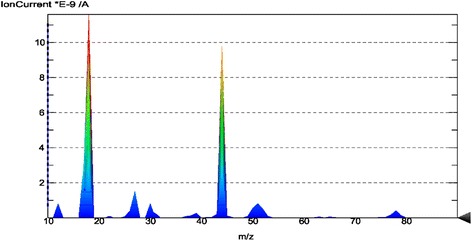
QMS-2D of the synthesized composite

The weight loss of the composite was identified in the range of 100 to 600 °C in accordance with thermogravimetric analysis data (Fig. 3). In particular, at 110°–120°, a negligible weight loss (1.24 wt.%) was observed. Comparison of thermal analysis data with mass spectral data (Figs. 4 and 5) confirmed that such weight loss took place due to water desorption from the silica gel. The overall weight loss was at around 27 wt.%. A total amount of organic part in the composite was estimated at 25.73 wt.%.

The first stage of decomposition of composite polymer part was detected at 150–200 °C with maximum at 174.2 °C (3.68 wt.%). In the range 200–400 °C the weight loss was 10.31 wt.% with maximum at 331.5 °C. The major products in accordance with mass-spectroscopy data were aromatic fragments of styrene and pyridine with the relative mass of 77–79 Da, CH and CH_2_ fragments with corresponding relative mass 30, and 38 Da as well as N_2_ and CO with relative mass 28 Da. A final polymer destruction was observed at the temperature 500–600 °C with a maximum at 515.4 °C and weight loss 11.74 wt.%. MS analysis confirmed the formation of CO_2_ and N_2_O (44 Da) at this stage.

**Fig. 6:**
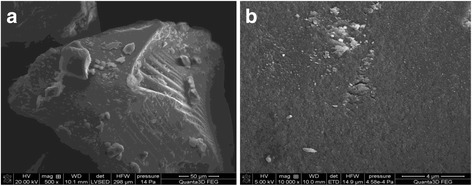
SEM-microphotographs of synthesized composite at ×500 (**a**) and ×10.000 (**b**) magnification

In order to clarify the geometry and location of the copolymer on the silica gel surface SEM-microphotographs of synthesized composite at 500 × (*A*), 10 000 × (*B*) magnification (Fig. 6) were obtained. As one can see, the immobilized polymer located on the surface of silica gel irregularly, “islet” and predominantly in the form of coils (globules) and their aggregates.

### Influence of pH on Adsorption

As we believed, such composite could exhibit an adsorption activity towards metal ions mainly due to the complexation with pyridine Nitrogen. To confirm this assumption, the adsorption of a such metal ions as Cu(II), Fe(III), and Ni(II) with high affinity to Nitrogen-containing ligands [10] was estimated for the composite in comparison with parent silica gel. In order to clarify the possibilities of practical use, we also studied the adsorption of some toxic metals ions, e.g., Cd(II), Pb(II), and Mn(II).

The dependence of adsorption of Cu(II), Cd(II), Pb(II), Mn(II), Fe(III), and Ni(II) ions by 4-vinylpyridine-styrene copolymer, in situ immobilized on silica gel surface, from acidity of the medium in the static mode is presented in Table 1.

**Table 1:** The degree of adsorption of Cu(II), Cd(II), Pb(II), Fe(III), Ni(II), and Mn(II) ions by 4-vinylpyridine-styrene copolymer, in situ immobilized on silica surface, as a function of the medium acidity

Ions	Degree of adsorption (*R*, %)				
	pH 1.0 HCl	pH 1.7 oxalate buffer	pH 4.0 phthalate buffer	Distilled water	pH 8.4 ammonium acetate buffer
Cu(II)	10.26	30.23	34.78	63.09	50.70
Cd(II)	4.65	13.19	24.35	95.38	36.82
Pb(II)	56.61	100.00	58.54	85.65	59.19
Fe(III)	11.69	20.00	22.32	100.00	37.03
Ni(II)	39.54	80.33	63.03	100.00	66.17
Mn(II)	3.41	11.50	6.17	60.63	25.85

Experimental conditions: mass of sorbent—0.1 g, volume of solution—25 mL, m^0^_M_—100 μg

In accordance with data obtained, composite revealed the best adsorption properties towards microquantities of Cu(II), Cd(II), Pb(II), Fe(III), Ni(II), and Mn(II) ions in an aqueous medium (without adding any buffer solutions). The composite totally absorbs microquantities of Fe(III) and Ni(II) ions and effectively removes all other metal ions in comparison with acidic and alkaline medium. A quantitative adsorption of ions Pb(II) at pH 1.7 against oxalate buffer was also obtained.

### Adsorption Capacity of Synthesized Composite in the Static Mode

In order to establish the values of the adsorption capacity of silica gel with immobilized copolymer 4-vinylpyridine-styrene towards Cu(II), Cd(II), Pb(II), Fe(III), and Ni(II) ions adsorption isotherms were measured. The isotherms were obtained for aqueous solutions of nitrates of these metals (without adding any buffer solutions) in static mode and compared with the initial silica gel. Taking into account that the lowest adsorption capacity was found towards ions of Mn(II), these ions were not examined.

It was found that the adsorption isotherm of the studied metals belongs to two different types. Adsorption isotherms Cu(II), Pb(II), and Ni(II) are 2L-type. This result is an evidence of the uniform distribution of adsorbed ions on the composite surface. The shape of Fe(III) and Cd(II) isotherms is typical for silica matrix modified by complexing substances [11]. The isotherms obtained were employed for calculation of the adsorption capacity with respect to each ion.

The adsorption capacity of the synthesized composite in comparison with parent silica gel for Cu(II), Pb(II), Ni(II) and Fe(III) ions calculated from isotherms are shown in Table 2.

**Table 2:** Adsorption capacity of silica surface, in situ immobilized by 4-vinylpyridine-styrene copolymer towards of Cu(II), Pb(II), Fe(III), and Ni(II) ions in the static mode

Ion	Adsorption capacity			
	Silica		Synthesized composite	
	mmol/g	mg/g	mmol/g	mg/g
Fe(III)	0.008	0.45	0.027	1.53
Pb(II)	0.002	0.41	0.075	15.54
Ni(II)	0.008	0.47	0.074	4.34
Cu(II)	0.005	0.32	0.029	1.84

Experimental conditions: mass of sorbents—0.1 g, volume of solution—25 mL

As follows from the data presented in the table, immobilization of silica gel surface led to increase the adsorption capacity towards Fe(III) ions in 3.4 times, Cu(II) ions in 5.8 times, Ni(II) ions in 9.2 times, and Pb(II) ions in 37.5 times. Thus, 4-vinylpyridine-styrene copolymer is promising material for waste water treatment from heavy metals, particularly when immobilized on the natural mineral carriers.

### Investigation of Ni(II) Ion Adsorption in the Cynamic Mode

An investigation of Ni(II) ions adsorption with initial concentration of their nitrate solutions 2–40 mg/l in dynamic mode revealed quantitative removal of these ions in all investigated concentration ranges. The data obtained showed a high rate of ions removal, what is typical for silica gels with immobilized on their surface complexing substances.

## Conclusions

In situ immobilization of 4-vinylpyridine-styrene copolymer on the surface of silica gel was performed by heterogeneous polymerization. The immobilization of copolymer was confirmed by IR and MS-spectroscopy. According to TG-analysis, the composite contained 25.73 wt.% of immobilized copolymer. Analysis of the SEM-microphotographs of synthesized composite showed that the immobilized copolymer located on the surface of silica gel not regularly, “islet” and predominantly in the form of coils (globules) and their aggregates.

Quantitative adsorption of microquantities of Fe(III) and Ni(II) ions and removing more than 1/2 of the original mass of metal ions Cu(II), Cd(II), Pb(II), and Mn(II) from aqueous solutions of nitrates in static mode adsorption was established. Quantitative adsorption at the same conditions of Ni(II) ions in the concentration range 2-40 mg/l in dynamic mode was discovered, too.

Immobilization of copolymer on silica gel surface resulted in improving its absorption capacity towards ions Fe(III) in 3.4 times, Cu(II) in 5.8 times, Ni(II) in 9.2 times, and Pb(II) 37.5 times.

The obtained results showed that the immobilization of small amounts of copolymers on the surface of natural mineral matrix could be a promising approach to developing effective adsorbents of heavy metals ions from aqueous solutions.

## Abbreviations

AIBN: 2,2′-Azobis(2-methylpropionitrile)
DSC: Differential scanning calorimetry
DTG: Differential thermo-gravimetry
IR: Infrared
MS: Mass-spectroscopy
QMS: Quadrupole mass-spectroscopy
SEM: Scanning electron microscopy
TG: Thermogravimetry
